# T-cell autoreactivity to citrullinated autoantigenic peptides in rheumatoid arthritis patients carrying HLA-DRB1 shared epitope alleles

**DOI:** 10.1186/ar3848

**Published:** 2012-05-17

**Authors:** Soi Cheng Law, Shayna Street, Chien-Hsiung Alan Yu, Christelle Capini, Sakoontalla Ramnoruth, Hendrik J Nel, Eline van Gorp, Claire Hyde, Kim Lau, Helen Pahau, Anthony W Purcell, Ranjeny Thomas

**Affiliations:** 1 The University of Queensland Diamantina Institute, Princess Alexandra Hospital, Brisbane, Qld 4102, Australia; 2 Department of Biochemistry and Molecular Biology, The Bio21 Molecular Science and Biotechnology Institute, University of Melbourne, Parkville, Vic 3010, Australia

## Abstract

**Introduction:**

Anti-citrullinated peptide antibodies are found in rheumatoid arthritis (RA) patients with HLA-DRβ chains encoding the shared epitope (SE) sequence. Citrullination increases self-antigen immunogenicity, through increased binding affinity to SE-containing HLA-DR molecules. To characterise T-cell autoreactivity towards citrullinated self-epitopes, we profiled responses of SE^+ ^healthy controls and RA patients to citrullinated and unmodified epitopes of four autoantigens.

**Methods:**

We compared T-cell proliferative and cytokine responses to citrullinated and native type II collagen 1,237 to 1,249, vimentin 66 to 78, aggrecan 84 to 103 and fibrinogen 79 to 91 in six SE^+ ^healthy controls and in 21 RA patients with varying disease duration. Cytokine-producing cells were stained after incubation with peptide in the presence of Brefeldin-A.

**Results:**

Although proliferative responses were low, IL-6, IL-17 and TNF were secreted by CD4^+ ^T cells of SE^+ ^RA patients and healthy controls, as well as IFNγ and IL-10 secreted by RA patients, in response to citrullinated peptides. Of the epitopes tested, citrullinated aggrecan was most immunogenic. Patients with early RA were more likely to produce IL-6 in response to no epitope or to citrullinated aggrecan, while patients with longstanding RA were more likely to produce IL-6 to more than one epitope. Cytokine-producing CD4^+ ^T cells included the CD45RO^+ ^and CD45RO^- ^and the CD28^+ ^and CD28^- ^subsets in RA patients.

**Conclusion:**

Proinflammatory cytokines were produced by CD4^+ ^T cells in SE^+ ^individuals in response to citrullinated self-epitopes, of which citrullinated aggrecan was most immunogenic. Our data suggest that the T-cell response to citrullinated self-epitopes matures and diversifies with development of RA.

## Introduction

Rheumatoid arthritis (RA) is an autoimmune disease characterised by inflammation of joint synovial tissue and deformity and destruction of associated bone, cartilage and soft tissues. Several autoantigens are described in RA, including a variety of proteins that become citrullinated in diseased joints. Citrullination is a physiological process of arginine deimination that occurs during apoptosis and inflammation. This process results in modification of arginine-containing proteins, which can give rise to sets of neo-self-antigens in individuals bearing at-risk HLA alleles [1].

Specific HLA-DR gene variants mapping to amino acids 70 to 74 of the third hypervariable region of DRβ chains are highly associated with RA [2]. This region encodes a conserved amino acid sequence that forms the fourth anchoring pocket (P4) in the HLA-DR antigen-binding groove. This shared susceptibility epitope (SE) is found in multiple RA-associated DR alleles, including DRB1*0401, DRB1*0404 and DRB*0101 in Caucasians [2]. The SE-encoding HLA alleles are particularly associated with anti-citrullinated protein autoantibody (ACPA)-positive RA [3-5]. The SE is highly positively charged, and is situated in a region of the DRβ chain that influences the specificity of the P4 amino acid of the bound ligand. The SE would therefore preferentially bind peptides containing a negatively charged or nonpolar amino acid at this position.

Citrullination replaces charged arginine amino side-chain groups with an uncharged carbonyl group, and has been shown to be permissive of binding of a human vimentin peptide epitope to SE^+ ^HLA DR molecules through increased affinity with P4 [6,7]. While the dominant B-cell vimentin, fibrinogen and collagen type II epitopes in RA are citrullinated, evidence is just emerging that T cells show specificity towards citrullinated over the corresponding native epitopes [7].

Approximately 70% of RA patient sera contain ACPAs [8]. This reactivity reflects autoantibody production towards a group of citrullinated autoantigens modified post-translationally, including fibrinogen, vimentin, collagen type II and enolase [9]. ACPAs develop up to 15 years prior to the onset of RA, with increasing titres and peptide reactivities as disease onset becomes imminent [10]. Citrullinated proteins have been demonstrated in inflamed tissues in RA, and ACPAs are induced in a number of mouse models of inflammatory arthritis [11-14]. Although citrullination is ubiquitous in response to stress and inflammation, ACPAs are highly specific for RA and are associated with more severe joint damage and radiographic outcome [3,4,8].

Immunisation of HLA-DRB1*0401 transgenic mice with citrullinated fibrinogen, but not native fibrinogen, induced inflammatory arthritis characterised by simultaneous B-cell and T-cell autoreactivity to citrullinated and native HLA-DR-restricted fibrinogen epitopes, which was not present in naïve HLA-DRB1*0401 transgenic mice [15]. Furthermore, recent studies suggest that delivery of the costimulation modulator, abatacept, may restore tolerance towards citrullinated antigens [16]. Notwithstanding these studies in transgenic mice, citrulline-specific autoreactive T cells have been difficult to demonstrate in RA patients due to the weak proliferative responses made by autoreactive effector memory T cells *in vitro*. However, several recent papers have shown convincing cytokine responses made by RA patient T cells in response to citrullinated vimentin and aggrecan epitopes [7,17]. In the case of vimentin, the immunogenicity of the epitope was dependent on the location of the citrulline modification within the peptide sequence [7].

In the current studies, we profiled the responses of SE^+ ^healthy controls and RA patients towards a set of citrullinated and unmodified (native) self-antigens, and characterised the responding T cells. Our aim was to identify citrullinated epitopes that may be of particular relevance in ACPA^+ ^RA, and to determine the extent of individual variability among citrullinated autoantigenic T-cell responses. Further, we wished to identify T cells that may contribute to the development of ACPA^+ ^RA. Of four citrullinated autoantigenic peptides, we identified citrullinated aggrecan 84 to 103 as the most immunogenic, and we identified IL-6 production by T cells as an important biomarker of response.

## Materials and methods

### Patients

Twenty-one patients who fulfilled the 1987 American College of Rheumatology criteria for RA [18] and six ACPA^- ^SE^+ ^healthy controls were included. All individuals provided peripheral blood (PB) samples, although in some cases the yield was insufficient for all assays. Patient demographic details are outlined in Table 1. HLA-DR genotyping was carried out at Queensland Health Pathology Services. The study was approved by the Human Research Ethics Committee of the Princess Alexandra Hospital and informed consent was obtained from each patient.

**Table 1:** Characteristics of patients in the study

Patient	Ethnicity	HLA-DRB1 genotype	ACPA	Disease duration (years)	Treatment	C-reactive protein^a ^(mg/l)	Representation
RA1	Caucasian	03,0401	+	6	M, S	8	Figure 1
RA2	Caucasian	03, 0401	+	< 1	Nil	3	Figures 1 to 5
RA3	Caucasian/Pacific Islander	0403, 0405	-	< 1	M, S, H	3	Figure 1
RA4	Caucasian	0401, 0404	+	< 1	L	11	Figures 1 to 5
RA5	Caucasian	0404, 1302	+	1	M, S, H	2	Figure 1
RA6	Caucasian	0401 13	+	> 5	M, H	1	Figures 1 to 5
RA7	Asian	0405	-	1	M	1	Figures 1 to 5
RA8	Caucasian	0401, 0404	+	> 5	L, H, S	22	Figures 1 to 5
RA9	Caucasian	0103, 0101	+	< 1	Nil	4	Figures 1 to 5
RA10	Caucasian	01, 1302	+	5	H	10	Figures 1 to 5
RA11	Caucasian	04, 0301	-	4	M, S, H	3	Figures 1 to 5
RA12	Caucasian	0401	+	0	Nil	45	Figures 1 to 5
RA13	Caucasian	0101, 1301	-	> 5	M	20	Figures 1 to 6
RA14	Caucasian	0408, 11	+	> 5	M, S, H	4.6	Figures 1 to 6
RA15	Caucasian	0401, 0408	+	0	Nil	1	Figures 1 to 6
RA16	Caucasian	0101, 0408	+	0	Nil	6	Figures 1 to 6
RA17	Caucasian	0401, 03	+	> 5	M, A	33	Figure 6
RA18	Caucasian	0401, 03	+	3	M	2	Figures 1 to 5
RA19	Caucasian	0101, 0103	+	2	M, S, H	5.3	Figures 1 to 5
RA20	Caucasian	0401, 1501	+	2	-	9.9	Figures 1 to 5
RA21	Caucasian	0401, 0101	+	> 5	M, P	2	Figures 1 to 5
HC1	Caucasian	01	-				Figures 1 to 5
HC2	Caucasian	03, 04	-				Figures 1 to 5
HC3	Caucasian	0401, 0701	-				Figures 1 to 5
HC4	Caucasian	0101, 08	-				Figures 1 to 5
HC5	Caucasian	1301, 0401	-				Figures 1 to 6
HC6	Caucasian	0401, 1301	-				Figures 1 to 6

A, abatacept; ACPA, anti-citrullinated peptide antibody; H, hydroxychloroquine; L, leflunomide; M, methotrexate; S, sulfasalazine; P, prednisone. For rheumatoid arthritis (RA) patients, mean age is 50 (range 22 to 65), 90% female; for healthy control (HC) individuals, mean age is 38 (40 to 45), 50% female. ^a^Measured at the time of blood withdrawal for peptide response.

### Peptide preparation

Citrulline or arginine-containing SE-binding epitopes were synthesised from vimentin, collagen type II, fibrinogen and aggrecan (Auspep Tullamarine VIC, Australia). All peptides were filtered, reconstituted to 300 μg/ml in sterile water and stored at -70°C. Final dilutions for the working stock were made with medium.

### Purification of peripheral blood mononuclear cells and antigen presentation assays

Peripheral blood mononuclear cells (PBMC) were isolated using Ficoll-Paque density gradients (GE Healthcare, Piscataway, New Jersey, United States), washed with 0.9% saline and then resuspended in RPMI containing 10% human serum from healthy or autologous or allogeneic RA donors. Tetanus toxoid was used at a concentration of 4 Lfi/ml (Chiron Vaccines, Mumbai, Maharashtra, India). Two × 10^5 ^PBMC or SFMC were incubated with 0, 3 and 30 μg/ml of each peptide in the presence of RPMI containing 10% human serum in a final volume of 200 μl in round-bottomed wells for 5 days. T-cell proliferation was assessed by addition of 1 μCi/well [^3^H]thymidine (ICN Biochemicals Costa Mesa, CA, USA) for the final 18 hours. Cells were harvested onto glass-fibre filter mats and [^3^H]thymidine incorporation was determined by liquid scintillation spectroscopy (Packard Topcount; Packard Instrument Co. Meridien, CT, USA). IL-2, IL-4, IFNγ, IL-10, IL-6, IL-17 and TNF were measured in the day 5 supernatants using BD Cytometric Bead Array kits (BD Bioscience, San Jose, CA, USA).

Initial kinetic experiments demonstrated that peptide stimulation for 5 days optimally induced antigen-specific proliferative and cytokine responses, and that longer cultures did not yield greater responses. Where RA serum was used in cytokine production assays, 150 μg/ml HeteroBlock (Omega Biologicals Bozeman, MT, USA) were added during BD Cytometric Bead Array measurement [19]. Samples were read on the BD FACSArray™ bioanalyser system.

Stimulation indices for proliferative responses were calculated as the fold increase in response to peptide over background. Net cytokine secretion was calculated for each response as the concentration with peptide stimulation minus the concentration without peptide stimulation. Positive response thresholds were two standard deviations above the cytokine responses towards the corresponding native peptide, for both RA patients and healthy controls.

### Flow cytometry and intracellular cytokine staining

PBMC from SE^+ ^RA patients or healthy controls were incubated without or with peptides at concentrations of 3 and 30 μg/ml for 5 days, with Brefeldin-A (Sigma Aldrich St Louis, MO, USA) added for the final 18 hours. Cells were stained for CD3-FITC, CD4-APC/Cy7, CD28-FITC, CXCR5-PerCP/Cy5.5 and CD45RO-PerCP/Cy5.5 (Biolegend, San Diego, CA, USA), followed by permeabilisation and staining for intracellular IL-6-APC and IFNγ-APC (Biolegend). Data were collected on the Gallios flow cytometer and analysed using Kaluza software (Beckman Coulter, Indianapolis IN, USA).

### Statistical analysis

One-way nonparametric analysis of variance with *post-hoc *correction (Kruskal-Wallis test) compared multiple means. Unpaired Mann-Whitney tests compared the tetanus toxoid proliferative responses between RA patients and healthy controls and compared specific cytokine responses with citrullinated versus native peptides. Significance is indicated as *P *< 0.05, *P *< 0.01 and *P *< 0.001. All error bars represent the standard error of the mean.

## Results

### T cells proliferate poorly but produce cytokines in response to citrullinated autoantigenic peptides

**Figure 1:**
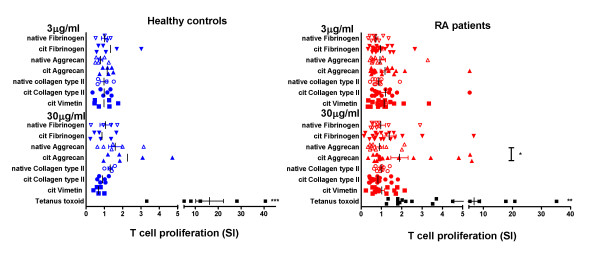
**T-cell proliferative response of healthy controls and rheumatoid arthritis patients stimulated with peptides**. Peripheral blood mononuclear cells (PBMC) from 20 rheumatoid arthritis (RA) patients and six healthy controls were incubated with 0, 3 or 30 μg/ml citrullinated (cit) and native peptides or 4 Lfi/ml tetanus toxoid, as shown, for 5 days. T-cell proliferation was assessed by uptake of [^3^H]thymidine. Each dot represents one individual. ***P < 0.001 for RA patients and **P < 0.01 for healthy controls comparing multiple means (Kruskal-Wallis test). **P < 0.01 comparing healthy controls and RA patient PBMC for the response to tetanus toxoid (Mann-Whitney test). *P < 0.05 comparing RA patients' responses to citrullinated and native aggrecan peptides (Mann-Whitney test). SI, stimulation indices.

We synthesised citrullinated or unmodified peptide antigens from the fibrinogen, vimentin, collagen type II and aggrecan protein sequences that had been identified - either based on predicted binding capacity to RA-associated DR molecules in a molecular model positioning citrulline at P4, or through previous studies in HLA-DR4-IE-transgenic mice (Table 2) [6,15,17]. In total, we studied 21 SE^+ ^RA patients and six SE^+ ^healthy controls. All except four RA patients were also ACPA^+ ^(Table 1). Forty-three per cent of the RA patients were nonsmokers, 38% past smokers and 19% current smokers. One healthy control was a current smoker, and two controls had a family history of RA. We analysed the proliferative response of PBMC from SE^+ ^RA patients and SE^+ ^healthy controls to varying concentrations of citrullinated or unmodified peptide antigen. Proliferative responses to peptide antigens and tetanus toxoid were expressed as stimulation indices. The mean proliferative stimulation indices to citrullinated and unmodified peptides were generally between 1 and 2 in RA patients and healthy controls, and were significantly lower in each case than the responses to tetanus toxoid (Figure 1). The proliferative stimulation indices in response to citrullinated aggrecan peptide were significantly higher than those to native aggrecan peptide in RA patients (Figure 1). Proliferative responses to tetanus toxoid were significantly lower when comparing RA PBMC with healthy control PBMC.

**Table 2:** Sequences of peptides used in the study

Native peptide sequence	Citrullinated peptide sequence	Human native protein
QDFTNRINKLKNS	QDFTNCitINKLKNS	Fibrinogen-α 79 to 91
VVLLVATEGRVRVNSAYQDK	VVLLVATEGCitVRVNSAYQDK	Aggrecan 84 to 103
SAVRARSSVPGVR	SAVRACitSSVPGVR	Vimentin 66 to 78
QYMRADQAAGGLR	QYMCitADQAAGGLR	Collagen II 1,237 to 1,249

**Figure 2:**
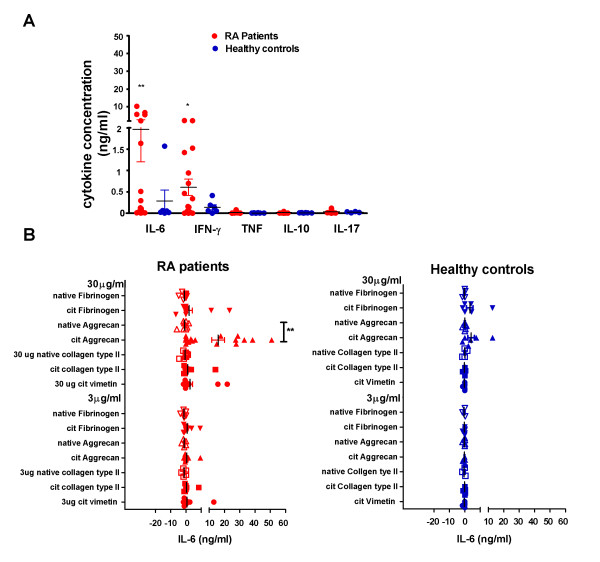
**Unstimulated cytokine secretion and net IL-6 secretion by peripheral blood mononuclear cells stimulated with peptides**. **(A) **Peripheral blood mononuclear cells (PBMC) from 17 rheumatoid arthritis (RA) patients and six healthy controls were incubated in medium for 5 days and cytokines were assessed in supernatants by the BD Cytometric Bead Array. **P < 0.01, *P < 0.05 (Kruskal-Wallis test with *post-hoc *correction). (**B) **PBMC from 17 RA patients and six healthy controls were incubated with 0, 3 or 30 μg/ml citrullinated (cit) and native peptides as shown, and IL-6 was assessed in supernatants by the BD Cytometric Bead Array. Net cytokine secretion was calculated as the IL-6 concentration with peptide stimulation minus the IL-6 concentration without peptide stimulation. Each dot represents one individual. *P < 0.05, **P < 0.01 comparing IL-6 responses with citrullinated and native peptides (Mann-Whitney test).

In initial experiments we determined that background cytokine secretion and proliferative responses were generally lower if peptide responses were assayed in the presence of human rather than foetal calf serum. Moreover, there was no difference in cytokine production when comparing assays carried out in the presence of 10% healthy or autologous or allogeneic RA donor serum, provided HeteroBlock was added when rheumatoid factor titres were > 100 U/ml (data not shown) to prevent rheumatoid factor from binding capture and detection antibodies in ELISA reactions [19]. In the absence of peptide stimulation, RA patient PBMC secreted significantly higher concentrations of IFNγ and IL-6 than of TNF and IL-10, secreted by either RA patients or healthy controls in the absence of peptides (Figure 2A). Net cytokine secretion was estimated as cytokine secreted upon stimulation with citrullinated peptides minus cytokine secreted in the absence of peptides. IL-6 secretion was highest of the cytokines measured, with net production of up to 60 ng/ml in response to 30 μg/ml citrullinated aggrecan.

**Figure 3:**
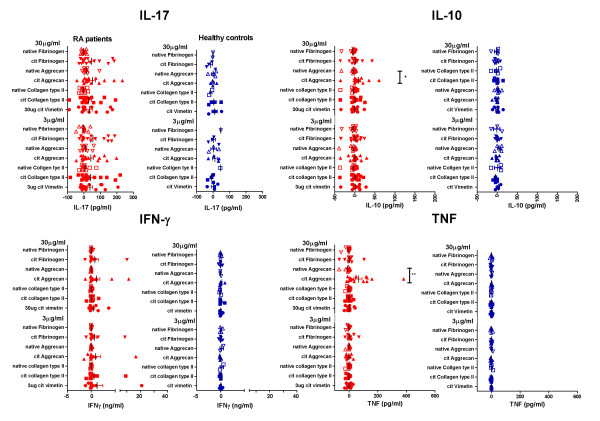
**Net cytokine secretion by peripheral blood mononuclear cells stimulated with citrullinated and native peptides**. Net TNF, IFNγ, IL-10 and IL-17 secretion by peripheral blood mononuclear cells from 17 rheumatoid arthritis (RA) patients and six healthy controls incubated with 0, 3 or 30 μg/ml citrullinated (cit) and native peptides as shown. Cytokines were assessed in supernatants by the BD Cytometric Bead Array. Net cytokine secretion was calculated as in Figure 2B. Each dot represents one individual. *P < 0.05, comparing IL-10 responses with citrullinated and native aggrecan (Mann-Whitney test).

Consistent with the lack of binding of native epitopes to shared epitope HLA-DR alleles [7] (and unpublished data), RA patients produced significantly greater amounts of IL-6, TNF and IL-10 in response to citrullinated than native aggrecan peptides (Figures 2B and 3). IL-17 and to some extent IFNγ were also secreted in response to several citrullinated peptides, but there was considerable variability among individuals and none of the differences was statistically significant (Figure 3). IL-2 and IL-4 responses were generally low (data not shown). When comparing RA patients and healthy controls, RA patients secreted significantly more IL-10 and TNF in response to citrullinated aggrecan than healthy controls (*P *< 0.05), and there was a similar trend for IL-17 secretion in response to citrullinated aggrecan (*P *= 0.065) and citrullinated fibrinogen (*P *= 0.08).

**Figure 4:**
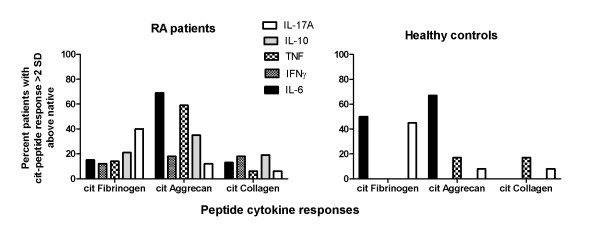
**Representation of cytokines produced by rheumatoid arthritis patients and healthy controls**. The percentage of rheumatoid arthritis (RA) patients or of healthy controls with positive responses (> 2 standard deviations (SD) above the mean response towards the corresponding native peptide) was calculated for each cytokine, and is plotted for responses to citrullinated (cit) fibrinogen, aggrecan and collagen type II.

Given the lack of stable HLA-DR binding of native self-peptides, we determined positive responses to citrullinated peptides to be greater than a threshold of the mean and two standard deviations above responses to the corresponding native peptides (citrullinated vimentin responses could not be assessed as responses to native vimentin were not measured). When compared with SE^+ ^healthy controls, SE^+ ^RA patients' citrullinated peptide responses produced a more diverse array of cytokines. Notably, when the percentage of RA patients and healthy controls with positive responses to each citrullinated peptide was plotted, the regulatory cytokines IL-10 and IFNγ were only produced by RA patients for the tested epitopes (Figure 4).

### IL-6 response among RA patients varies with disease duration

**Figure 5:**
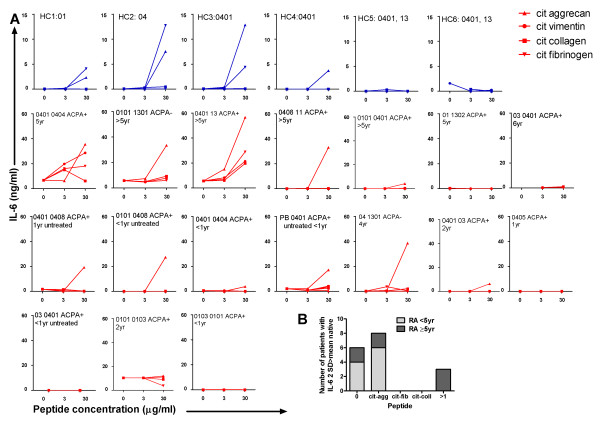
**Diversity of citrullinated peptide-reactive IL-6 response among rheumatoid arthritis patients and healthy controls**. **(A) **Peripheral blood mononuclear cells from 17 rheumatoid arthritis (RA) patients and six healthy controls were incubated with 0, 3 or 30 μg/ml citrullinated (cit) and native peptides as shown, and each individual's IL-6 response in supernatant was plotted. Disease duration and HLA type are indicated. **(B) **The frequency of RA patients with either recent-onset or longstanding disease with positive responses (> 2 standard deviations (SD) above the mean response towards the corresponding native peptide) for each peptide is shown. ACPA, anti-citrullinated peptide antibody.

To better understand the citrullinated peptide response pattern of individual RA patients and SE^+ ^healthy controls, we plotted IL-6 dose-response curves for each peptide for each individual in the study. PBMC from four out of six healthy controls dose-dependently secreted IL-6 to citrullinated aggrecan and PBMC from three out of six controls secreted IL-6 in response to citrullinated fibrinogen. Taking the same threshold for a positive response as described above, we found that six out of 17 RA patients' PBMC responses secreted IL-6 in response to no epitopes, eight patients' responses to citrullinated aggrecan only, none to citrullinated fibrinogen, none to citrullinated collagen type II only and three patients' responses to > 1 citrullinated epitope. IL-6 responses were thus highest and observed most frequently towards citrullinated aggrecan in both RA patients and healthy controls, suggesting this was the most immunogenic epitope tested. An IL-6 response to > 1 citrullinated epitope occurred more frequently among patients diagnosed with RA at least 5 years previously (Figure 5B).

### Effector memory CD4^+ ^T cells secrete cytokines in response to citrullinated peptides

**Figure 6:**
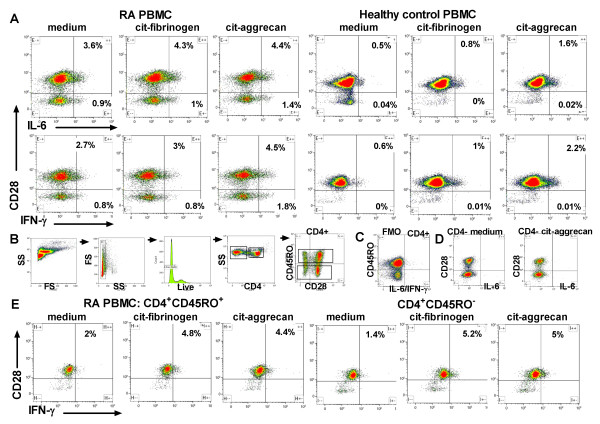
**Cytokine secretion by CD4^+ ^T-cell subsets**. Peripheral blood mononuclear cells (PBMC) from HLA-DR SE^+ ^rheumatoid arthritis (RA) patients and healthy controls were incubated with 0 or 30 μg/ml citrullinated (cit) fibrinogen or citrullinated aggrecan, stained with mAb directed against **(A) **CD4, CD28, IFNγ and IL-6 or **(E) **CD4, CD45RO, IL-6 and IFNγ, and then analysed by flow cytometry. **(B) **The gating strategy is outlined. FS, forward scatter; SS, side scatter. **(C) **Fluorescence minus one (FMO) plot showing background staining for intracellular cytokine staining, gated on CD4^+ ^T cells. **(D) **IL-6 staining of gated CD4^- ^non-T cells. The proportions in (E) of IL-6^+^CD4^+^CD45RO^+ ^T cells were 2.2%, 5.3% and 7.1%, and of IL-6^+^CD4^+^CD45RO^- ^T cells were 0.8%, 2.1% and 4% in response to incubation with no peptide, citrullinated fibrinogen and citrullinated aggrecan, respectively. Two individual RA patients and one healthy control are shown. Data are representative of two healthy donors and five RA patients.

IL-6 is an important cytokine in RA, which could be produced upon stimulation of PBMC by either T cells or antigen-presenting cells. To determine the origin of the cytokines secreted into supernatants of PBMC stimulated by citrullinated peptides, we incubated RA PBMC with citrullinated or native peptides for 5 days, and with addition of Brefeldin-A for the last 18 hours, prior to intracellular cytokine staining along with analysis of cell surface markers. CD3^+^CD4^+ ^T cells produced more intracellular IFNγ and IL-6 when incubated with citrullinated aggrecan or fibrinogen relative to incubation in medium alone (Figure 6A, B).

Fluorescence minus one staining demonstrates the gating strategy to determine the threshold for positive staining, as described elsewhere [20] (Figure 6C). No intracellular cytokine staining was observed in these assays by CD4^-^CD28^- ^cells, most of which represent antigen-presenting cells (Figure 6D). Differentiated or ageing CD45RO^- ^memory cells have been shown to re-express CD45RA and to be characterised by the loss of CD27 and CD28 [21]. In healthy controls, CD28^- ^cells comprised only a small proportion of the CD4^+ ^T cells, and cytokine-secreting cells were exclusively CD4^+^CD28^+^. CD28^- ^cells were more abundant among CD4^+ ^PB T cells in some, but not all, RA patients. Where CD4^+^CD28^- ^T cells were present, IL-6 and IFNγ were secreted by both CD28^- ^and CD28^+^CD4^+ ^T cells in response to citrullinated peptides. We noted high background cytokine secretion in these cultures, as we had observed earlier in analysis of supernatants (Figures 2A and 6A). IFNγ^+^CD4^+ ^and IL-6^+^CD4^+ ^T cells included both CD45RO^+ ^and CD45RO^- ^cells (Figure 6E). CXCR5^+ ^follicular helper T cells did not express IL-6 or IFNγ in response to citrullinated peptides (data not shown).

## Discussion

We have shown here that proinflammatory and regulatory cytokines, including IL-6, IFNγ IL-10 and TNF, were produced by CD4^+ ^T cells in SE^+ ^RA patients in response to citrullinated self-epitopes, of which citrullinated aggrecan was most immunogenic. These cytokine responses were observed in spite of weak peptide-specific T-cell proliferative responses. SE^+ ^healthy controls also produced cytokines in response to citrullinated aggrecan and citrullinated fibrinogen. Such T-cell responses are not necessarily causally related to RA - rather, they demonstrate the autoreactivity present in SE^+ ^individuals towards citrullinated self-peptides, even in the absence of ACPAs. Cytokine responses to citrullinated self-epitopes, however, were more diverse in RA patients than in healthy control individuals. Indeed, only RA patients secreted the regulatory cytokines IL-10 and IFNγ in response to these epitopes [22]. IFNγ production by PB and synovial T cells is well described [23], and IFN-regulated genes have been shown to be predictive of RA development in ACPA^+ ^patients with arthralgia [24]. Using intracellular staining we demonstrated IL-6 and IFNγ production by memory CD4^+ ^T cells but not by CD4^- ^antigen-presenting cells in these cultures, at least when examined after 5 days of peptide stimulation. These data demonstrate that CD4^+ ^T cells were capable of cytokine production but do not exclude the possibility that CD4^- ^antigen-presenting cells such as monocytes, B cells and dendritic cells could also produce these cytokines during the culture, which could be secreted into the supernatant. IL-6 responses increased dose-dependently in response to citrullinated peptides. Intracellular cytokine staining confirmed a broad cytokine response profile, consistent with the phenotype of memory CD4^+ ^T cells.

Various mechanisms could contribute to the low proliferative response exhibited by autoreactive T cells to self-peptides and tetanus toxoid antigen in RA. For instance, a number of studies implicate deficient signalling of RA T cells through the T-cell receptor-CD3 complex [25-31]. IL-2 might be rapidly consumed through binding to the high avidity CD25 receptor expressed by regulatory T cells and by effector memory T cells upon activation. This could limit availability of IL-2 for T-cell proliferation in tissue culture [32,33]. Furthermore, autoreactive T cells are subject to the influence of regulatory cells and cytokines, as demonstrated here [34,35]. On the other hand, effector memory cells produce high levels of proinflammatory and regulatory cytokines, and cytokine production more effectively interrogates highly differentiated autoreactive effector memory T cells in both humans and mice [36-38].

The proinflammatory cytokine IL-6 stimulates B-cell antibody production and plays a critical role in RA pathogenesis [39]. In RA patients, intracellular IL-6 and IFNγ were produced by CD28^+ ^and CD28^- ^and CD45RO^+ ^and CD45RO^- ^PB CD4^+ ^T cells. Consistent with the poor proliferative but good cytokine response of RA T cells in response to citrullinated peptides in the current studies, CD4^+^CD45RB^dim^CD27^- ^memory T cells from healthy donors were previously shown to proliferate poorly but to produce large amounts of IL-4 and IL-10 in response to mitogen, and to provide effective B-cell help for immunoglobulin production [40]. In contrast, typical CXCR5^+ ^follicular helper T cells, which have been shown to promote antibody production in lymphoid tissue *in vivo *[41], did not express cytokines in response to citrullinated peptides. Of interest, where CD28^- ^T cells were present in PB of RA patients, they also produced cytokine in response to citrullinated peptides. CD28^- ^T cells represent an important effector memory amplification response in the synovial environment, and have been shown to be more resistant to suppression by regulatory T cells [21,42]. Together our data support the hypothesis that effector memory T cells, reactive with a variety of citrullinated self-peptides and with the potential for B-cell help, circulate in PB of SE^+ ^individuals.

Previous studies in RA patients analysed the PB CD4^+ ^T-cell response to a single citrullinated peptide specificity. Proliferative and IL-17 CD4^+ ^T-cell responses to citrullinated aggrecan 84 to 103 were demonstrated in RA patients but not in healthy controls, demonstrating the immunogenicity of this citrullinated aggrecan epitope [17]. The unmodified aggrecan epitope is immunodominant in BALB/c mice immunised with aggrecan/proteoglycan [43]. This citrullinated aggrecan peptide was also the most immunogenic epitope in the current studies - with the highest frequency of responders, and with the highest magnitude of IL-6 responses. In longstanding RA patients, we also found robust cytokine production in response to citrullinated fibrinogen-α 79 to 91, which was shown to be the immunodominant epitope in HLA-DR4-IE transgenic mice immunised with citrullinated human fibrinogen [15].

Stimulation with the citrullinated vimentin 66 to 78 epitope produced much weaker cytokine responses in the current studies - as was observed by Snir and colleagues, who found that HLA-DRB1*0401^+ ^RA patient T cells produced a number of cytokines when incubated with citrullinated vimentin 59 to 78 but not citrullinated vimentin 66 to 78 [7]. In that study, IFNγ and TNF were expressed intracellularly by a small proportion of CD154^+^CD4^+ ^RA patient T cells in response to citrullinated but not native vimentin 59 to 78. The proportion of T cells staining for intracellular cytokine was much lower than in the current studies, in which we carried out staining 5 days after antigen stimulation and without a second restimulation. Anergy may possibly have been induced by restimulation with peptide after an initial 5-day culture with peptide - or, given their effector memory phenotype [44], few T cells were capable of re-expressing CD154 after prolonged *in vitro *culture. Similar to the current study, Snir and colleagues did not observe strong proliferative or IL-17A responses, unlike von Delwig and colleagues [17].

Differences in cytokine secretion may relate to the inclusion of healthy human serum or of HeteroBlock plus autologous serum in the current studies [19]. Finally, due to the hydrophobic amino acids at 84 to 88, the citrullinated aggrecan peptide was relatively insoluble in aqueous medium, which may also have affected the antigen purity, concentration and cellular uptake in different laboratories. The greater immunogenicity of citrullinated aggrecan possibly also relates to its length relative to the other peptides. Structural analysis of the HLA-DR binding and T-cell contact residues of these peptides will aid understanding of the T-cell responses observed.

We observed T-cell cytokine responses to citrullinated self-epitopes in SE^+ ^RA patients, whether ACPA^+ ^or not, as well as in ACPA^- ^healthy controls. HLA-DRB1*0401^+ ^healthy control T cells were also observed to produce cytokine in response to citrullinated vimentin 59 to 78 [7]. After immunisation of DR4-IE transgenic mice with citrullinated human fibrinogen, responses to unmodified native epitopes were stimulated in parallel with responses to citrullinated epitopes. From the previous and current analyses of HLA-DR binding and responses to unmodified epitopes, responses to citrullinated epitopes appear to be most specific to SE^+ ^RA PB. In contrast, the magnitude and diversity of the responses to citrullinated epitopes in healthy controls was unexpected. CD4^+ ^T cells in both SE^+ ^RA patients and HLA-DR4^+ ^healthy controls have reduced T-cell-receptor excision circles, overall telomere shortening, and reduced replicative capacity, which together imply an HLA-DR SE-associated reduction in T-cell input to the peripheral repertoire, an increased proliferative drive for naïve T cells towards peripheral self-antigens and a limited diversity of the TCR repertoire [45-47].

Together, studies in humans suggest that PB T cells in HLA-DR SE^+ ^individuals may be predisposed to autoreactivity towards self-antigens, including those modified by citrullination, potentially exposed during stress or proinflammatory settings, including joint trauma or smoking [3,48]. Furthermore, unlike T-cell autoreactivity, the development of B-cell autoreactivity towards citrullinated epitopes appears to be a significant checkpoint in the progression to RA. It has been proposed that infection (for example, with *Porphyromonas gingivalis *in patients with periodontitis) may be a critical trigger for progress through this checkpoint, potentially due to cross-reactivity with bacterial antigens, adjuvant effects of pathogen-associated molecular patterns, targeting of alternative antigen-presenting cells, or a combination of these [9]. Carbamylation of lysine residues to homocitrulline may be another factor enhancing the immunogenicity of RA autoantigens, including citrullinated antigens [49].

Our data suggest that RA patients with longstanding disease may be more likely to respond to more than one citrullinated epitope, whereas patients with recent-onset RA (including those previously untreated) appeared generally to respond either to no antigen or only to citrullinated aggrecan. These data suggest the possibility that epitope-spreading occurs as disease progresses. To confirm this hypothesis, a larger sample of RA patients would need to be studied, preferably longitudinally, along with peptide-MHC tetramer reactivity. Furthermore, it would be preferable to study a panel of the most immunogenic citrullinated epitopes, including the citrullinated aggrecan and fibrinogen α epitopes used here, and citrullinated vimentin 59 to 78 [7]. The assay developed here, in which T-cell cytokine production is interrogated in the presence of varying concentrations of a panel of citrullinated epitopes, is likely to be a useful biomarker to identify the most immunogenic peptide epitopes, and to correlate specific T-cell responses with corresponding ACPA fine specificities [50]. These assays would be optimally carried out in conjunction with analysis of tetramer-specific T cells. Future applications include comparison of serial samples of PBMC from pre-RA through to diagnosis [51], and effects of treatment. The analysis of PBMC from clinical trials of antigen-specific or nonspecific immunotherapy, such as abatacept, would also be of interest [16]. Finally, analysis of specific peptide reactivity may be beneficial for stratifying patients and for identifying appropriate epitopes for personalised antigen-specific immunotherapy.

## Conclusion

Proinflammatory and regulatory cytokines were produced by CD4^+ ^T cells in SE-positive individuals in response to citrullinated self-epitopes, of which citrullinated aggrecan was most immunogenic. Our data suggest that the T-cell response matures and diversifies with development of RA.

## Abbreviations

ACPA: anti-citrullinated peptide antibody; IFN: interferon; IL: interleukin; mAb: monoclonal antibody; P4: fourth anchoring pocket; PB: peripheral blood; PBMC: peripheral blood mononuclear cells; RA: rheumatoid arthritis; SE: shared epitope; TNF: tumour necrosis factor.

## Competing interests

The authors declare that they have no competing interests.

## Authors' contributions

SCL, SS, C-HAY, CC, SR, HJN, EvG, CH and KL designed the peptides and carried out immunoassays. HP coordinated the clinical data collection. SCL, SS, CC, AWP and RT conceived the study, participated in its design and coordination, and helped to draft the manuscript. All authors read and approved the final manuscript.
